# Different effects of menopausal hormone therapy on non-alcoholic fatty liver disease based on the route of estrogen administration

**DOI:** 10.1038/s41598-023-42788-6

**Published:** 2023-09-19

**Authors:** Sung Eun Kim, Ji-Song Min, Saemi Lee, Dong-Yun Lee, DooSeok Choi

**Affiliations:** Department of Obstetrics and Gynecology, Samsung Medical Center, Sungkyunkwan University School of Medicine, 81 Irwon-ro, Gangnam-gu, Seoul, 06351 Korea

**Keywords:** Endocrinology, Gastroenterology, Health care

## Abstract

The effects of menopausal hormone therapy (MHT) on non-alcoholic fatty liver disease (NAFLD) were compared based on the route of estrogen administration. The study included 368 postmenopausal women who received MHT for 12 months. Patients were divided into transdermal (n = 75) and oral (n = 293) groups based on the estrogen route. Changes in the prevalence of NAFLD were compared between the two groups before and after 12 months of MHT. In addition, differences in the progression of NAFLD after MHT based on the dose of estrogen and type of progestogen were evaluated in the oral group. After MHT, the prevalence of NAFLD decreased from 24 to 17.3% in the transdermal group but increased from 25.3 to 29.4% in the oral group. Little or no change was found in clinical characteristics and laboratory tests in the transdermal group during MHT. However, serum levels of total cholesterol and low-density lipoprotein cholesterol decreased and triglycerides and high-density lipoprotein cholesterol increased significantly in the oral group. Furthermore, changes in the prevalence of NAFLD were not significantly different based on the dose of estrogen or type of progestogen. Our findings indicate that transdermal estrogen can be beneficial in terms of NAFLD progression.

## Introduction

Non-alcoholic fatty liver disease (NAFLD) is characterized by extensive accumulation of fat and triglycerides in the liver and not caused by excessive alcohol or drug use^1,2^. NAFLD is the most common chronic liver disease, and the prevalence in postmenopausal women is greater than 20% worldwide^3–5^. Although the prevalence of NAFLD differs based on age, sex, menopausal status, region, time, diagnostic tool, and definition, the incidence of NAFLD continues to increase. Furthermore, NAFLD can progress to more serious conditions such as cirrhosis and hepatocellular carcinoma; however, an established treatment does not exist. Therefore, NAFLD has clinical importance and should be further investigated.

NAFLD occurs less frequently in premenopausal women than in men or postmenopausal women^6,7^, indicating that estrogen plays a protective role against the progression of NAFLD. Estrogen inhibits proliferation of stellate cells and fibrogenesis in the liver^8^ and reduces hepatic fibrosis^9^. In addition, estrogen depletion can lead to visceral fat accumulation, weight gain as well as elevated triglycerides and cholesterol^10,11^, which are associated with NAFLD progression. In aromatase-deficient knockout mice, supplementation of estrogen restored gene expression and β-oxidation and prevented steatosis similar to in wild-type mice^12^. Furthermore, the incidence of NAFLD was lower^13^ and the progression of liver fibrosis was prevented^14^ in menopausal hormone therapy (MHT) patients.

Because hepatic effects differ based on the route of estrogen administration, the effects of MHT on NAFLD can also differ between transdermal and oral MHT treatment. Transdermal MHT bypasses the first-pass metabolism of the liver and thus has less influence on lipid metabolism, especially triglycerides, an important factor associated with NAFLD^15,16^. However, the effects of MHT on the prevalence of NAFLD based on the route of estrogen administration have not been evaluated to date. In addition, the effects of estrogen dose or progestogen type, which can affect lipid profile^17^, should also be evaluated.

In the present study, the effects on NAFLD were compared between transdermal and oral MHT treatment for 12 months in postmenopausal women.

## Materials and methods

### Study subjects

All consecutive postmenopausal women who received MHT at the Menopause Clinic in Samsung Medical Center (Seoul, Korea) to relieve menopausal symptoms and who had a routine checkup in the health promotion center from January 2016 to December 2020 were considered for this retrospective cohort study. The MHT consisted of estrogen and progestogen for women with a uterus but only estrogen for women without a uterus.

The inclusion criteria for the study were as follows: postmenopausal women 45–60 years of age, regardless of type of menopause (natural or induced), postmenopausal women who used the same MHT regimen for at least 12 months and had undergone a general health screening including abdominal ultrasonography before and after MHT. Women were excluded for the following reasons: (1) use of tibolone for MHT; (2) changed the MHT regimen within 12 months; (3) had any other liver disease; (4) had positive serology for hepatitis B or C; (5) consumed more than 10 g of alcohol per day; (6) used any drugs that can significantly affect hepatic metabolism; (7) used any drugs that can affect body weight.

Finally, 368 women were included in this study for analyses and divided into two groups based on the route of estrogen administration: transdermal MHT (n = 75) and oral MHT (n = 293). The Institutional Review Board of Samsung Medical Center approved the study protocol and informed consent was waived because the medical records were retrospectively reviewed. All methods were performed in accordance with relevant guidelines and regulations.

### Definitions

Menopause was defined as at least 12 months of consecutive amenorrhea or elevated serum follicle-stimulating hormone level > 20 IU/L.

NAFLD was defined based on hepatic steatosis from abdominal ultrasound. Ultrasound was evaluated by experienced doctors. Fatty liver evaluation criteria included liver parenchyma echogenicity compared with kidney, deep attenuation, and vascular structures^18^. Radiologists classified ultrasonography findings into four categories^19,20^: normal, mild (normal visualization of the diaphragm and intrahepatic vessel borders and a diffuse slight increase in echogenicity in the hepatic parenchyma), moderate (slightly impaired visualization of the diaphragm and intrahepatic vessel borders and diffuse increase in echogenicity), or severe (poor or no visualization of the diaphragm and intrahepatic vessel borders and significant increase in echogenicity). Progression of NAFLD was defined as newly diagnosed NAFLD after MHT in women with no NAFLD at baseline or exacerbation of pre-existing NAFLD after MHT.

For oral MHT, a low estrogen dose was defined as ≤ 1 mg of 17β-estradiol or conjugated estrogen < 0.625 mg^21^ and a standard estrogen dose as 2 mg of 17β-estradiol or 0.625 mg of conjugated estrogen. Transdermal MHT was used with an equivalent dose of a standard oral MHT. Natural progestogen included only micronized progesterone, and synthetic progestogens included dydrogesterone, cyproterone acetate, drospirenone, and norethisterone acetate.

### Measurements

Baseline clinical characteristics including age, age at menarche, age at menopause, parity, and smoking history were obtained from medical records. History and medication for hypertension, type II diabetes mellitus, and dyslipidemia were also evaluated. Before and after MHT, height and weight were measured to one decimal place, and body mass index was calculated by dividing weight by the square of height. Waist circumference was measured at the thickest part of the abdomen. Blood pressure was measured with a standard mercury sphygmomanometer after rest.

Using standard methods for each biochemical parameter, laboratory tests were performed in the morning after overnight fasting before and after MHT. The reference ranges of the laboratory tests were as follows: aspartate aminotransferase (0–32 U/L), alanine aminotransferase (0–33 U/L), alkaline phosphatase (35–104 U/L), gamma-glutamyl transferase (6–42 U/L), total bilirubin (0–1.2 mg/dL), fibrinogen (182–380 mg/dL), total cholesterol (0–200 mg/dL), triglycerides (0–149 mg/dL), high-density lipoprotein cholesterol (50–200 mg/dL), low-density lipoprotein cholesterol (0–129 mg/dL), fasting blood glucose (74–109 mg/dL), insulin (2.6–24.9 µIU/mL), hemoglobin A1c (4.0–6.0%), uric acid (2.4–5.7 mg/dL), and C-reactive protein (0–0.5 mg/dL). Insulin resistance was evaluated with the homeostasis model assessment of insulin resistance (HOMA-IR) using the following equation: insulin resistance = fasting blood insulin (µU/mL) × fasting blood glucose (mg/dL)/405.

Abdominal ultrasound was performed on the liver, gallbladder, pancreas, and kidney by experienced radiologists using a Philips Ultrasonography (Bothell, WA, USA) 4-MHz probe.

### Statistical analysis

All statistical analyses were performed using SPSS version 27 (SPSS Inc, Chicago, IL, USA). Data are presented as the mean ± standard deviation or percentage. The assumption of normality was evaluated before statistical analysis. Clinical characteristics and laboratory results were compared between transdermal and oral MHT groups using *t* test for continuous variables and chi-square test for categorical variables. Changes in the clinical characteristics and laboratory results before and after MHT were assessed using paired *t* test within each treatment. In addition, progression of NAFLD based on ultrasonography was compared using the chi-square test based on the route of estrogen administration as well as the dose of estrogen and type of progestogen in the oral MHT group. The results were considered statistically significant when *P* values were < 0.05.

## Results

Table 1 shows the comparisons of baseline characteristics between the transdermal and oral MHT groups. The mean age was 54.3 years in the transdermal MHT group and 53.7 years in the oral MHT group. Age, reproductive or menstrual history, body mass index, blood pressure, and history of chronic medical diseases did not differ between the two groups before starting MHT.

**Table 1:** Baseline clinical characteristics of patients.

	Transdermal MHT (n = 75)	Oral MHT (n = 293)	*P* value
Age (years)	54.3 ± 3.4	53.7 ± 3.4	0.201
Parity (n)	1.8 ± 0.8	1.8 ± 0.7	0.738
Age at menarche (years)	13.6 ± 1.4	13.7 ± 1.5	0.680
Age at menopause (years)	49.2 ± 3.4	49.4 ± 3.7	0.794
Years since menopause	5.1 ± 4.1	4.4 ± 4.1	0.202
Body mass index (kg/m^2^)	22.5 ± 3.0	22.2 ± 2.8	0.475
Waist circumference (cm)	74.2 ± 7.2	74.5 ± 7.1	0.778
Systolic blood pressure (mmHg)	114.8 ± 14.6	112.6 ± 14.8	0.303
Diastolic blood pressure (mmHg)	70.3 ± 10.0	68.9 ± 10.8	0.366
Hypertension (%)	12.0	7.8	0.255
Type II Diabetes mellitus (%)	4.0	2.7	0.703
Dyslipidemia (%)	24.0	15.0	0.064
Current smoking (%)	2.7	2.4	1.000

Data are presented as mean ± standard deviation or percent. *P* value based on *t* test or chi-square test, as indicated.

*MHT* menopausal hormone therapy.

**Figure 1:**
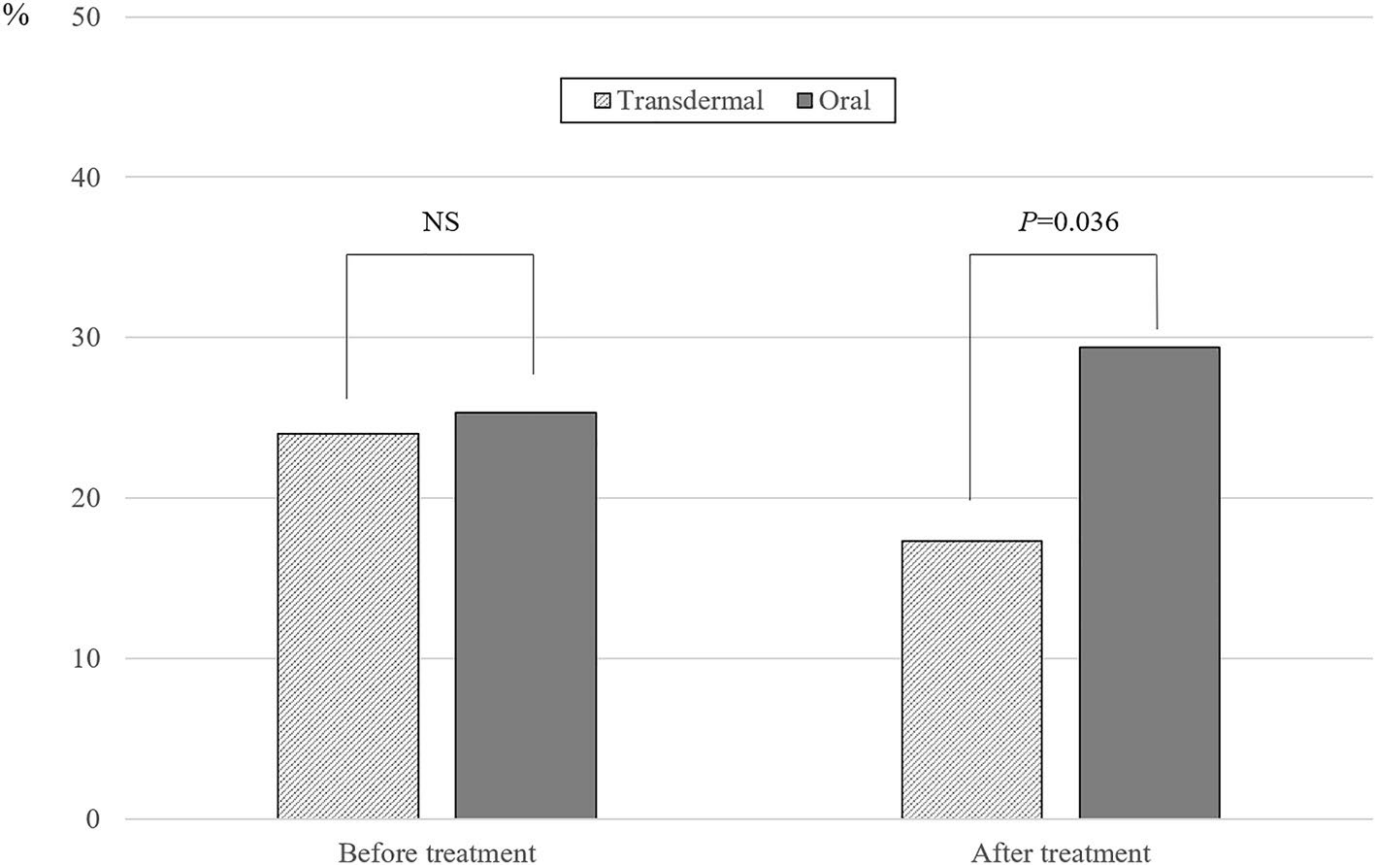
The prevalence of non-alcoholic fatty liver disease (NAFLD) before and after menopausal hormone therapy (MHT) based on the route of estrogen administration. *P* value based on chi-square test. NS, non-significant.

**Figure 2:**
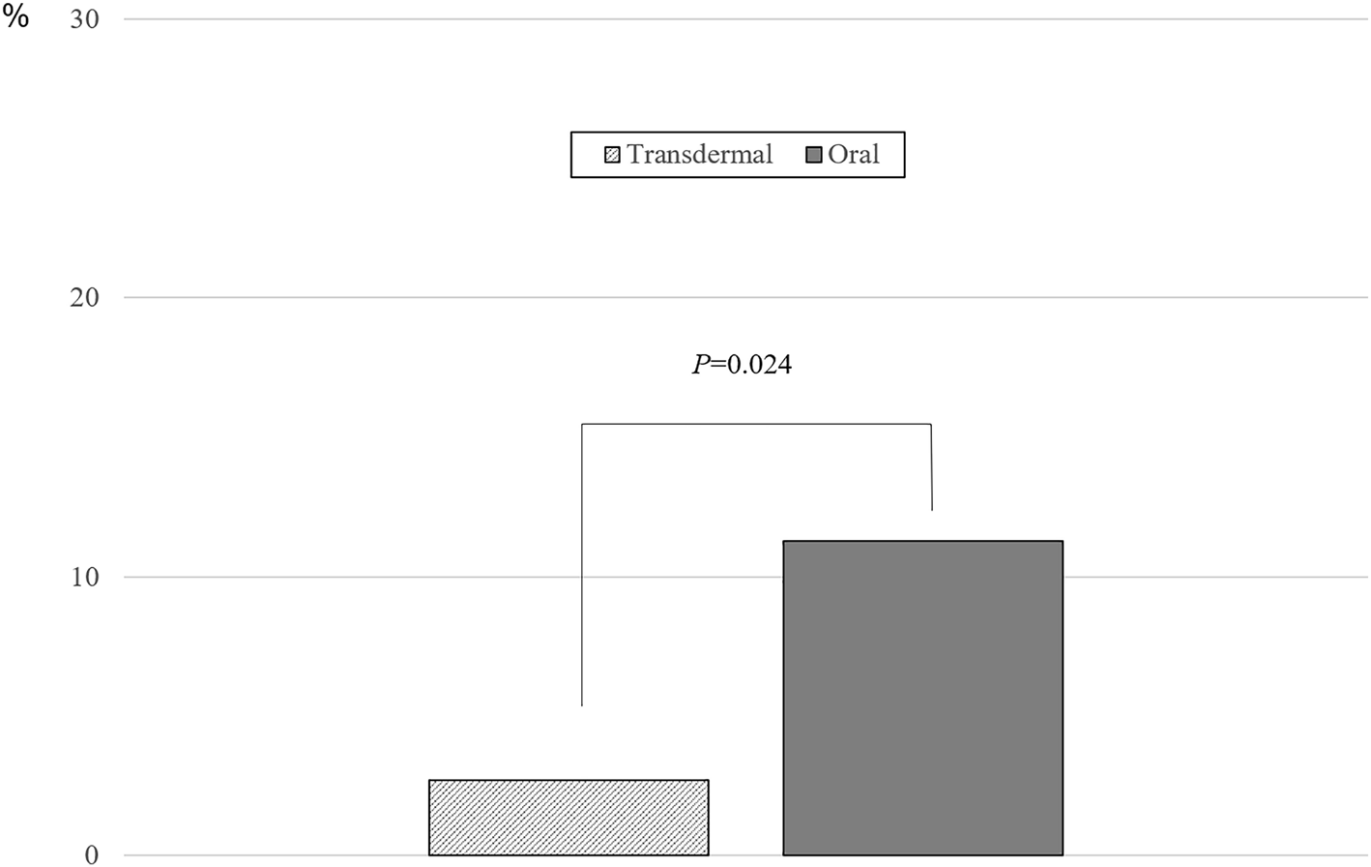
The proportion of progression (newly diagnosed or worsened severity) in non-alcoholic fatty liver disease (NAFLD) after menopausal hormone therapy (MHT) based on route of estrogen administration. *P* value based on chi-square test.

The prevalence of NAFLD before and after MHT based on the route of estrogen administration is shown in Fig. 1. Among 368 postmenopausal women, 24.7% (24.0% in the transdermal MHT group and 25.3% in the oral MHT group) had pre-existing NAFLD before MHT; however, a difference was not found in the prevalence based on the route of estrogen administration. After 12 months of MHT, the prevalence of NAFLD was 17.3% in the transdermal MHT group and 29.4% in the oral MHT group with statistically significant difference (*P* = 0.036). In addition, when the progression of NAFLD after MHT (newly diagnosed NAFLD or worsened pre-existing NAFLD) was compared based on the route of estrogen administration, the proportion of progression was significantly lower in the transdermal MHT group than in the oral MHT group (2.7% vs. 11.3%, *P* = 0.024; Fig. 2). In addition, progression after MHT was more prominent in women with pre-existing NAFLD (16.2%) than without NAFLD (9.6%) in the oral MHT group but did not reach statistical significance (data not shown).

Tables 2 and 3 show the changes in clinical characteristics and laboratory results during 12 months of MHT. In the transdermal MHT group, significant changes were not found in most variables during MHT, except a decrease of diastolic blood pressure. However, in the oral MHT group, serum levels of total cholesterol and low-density lipoprotein cholesterol decreased, and triglycerides and high-density lipoprotein cholesterol significantly increased after MHT. In addition, serum fibrinogen level was significantly increased after MHT in the oral MHT group.

**Table 2:** Changes in clinical characteristics after 12 months of MHT.

	Transdermal MHT (n = 75)		Oral MHT (n = 293)	
	Baseline	12 months	Baseline	12 months
Body mass index (kg/m^2^)	22.5 ± 3.0	22.5 ± 2.9	22.2 ± 2.8	22.1 ± 2.5
Waist circumference (cm)	74.2 ± 7.2	74.4 ± 7.1	74.5 ± 7.1	74.2 ± 7.2
Systolic blood pressure (mmHg)	114.8 ± 14.6	112.2 ± 13.8	112.6 ± 14.8	113.1 ± 15.3
Diastolic blood pressure (mmHg)	70.3 ± 10.0	67.6 ± 9.4*	68.9 ± 10.8	68.5 ± 11.4

Data are presented as mean ± standard deviation.

*P < 0.05 based on paired *t* test within each treatment.

*MHT* menopausal hormone therapy.

**Table 3:** Changes in laboratory results after 12 months of MHT.

	Transdermal MHT (n = 75)		Oral MHT (n = 293)	
	Baseline	12 months	Baseline	12 months
AST	20.9 ± 6.7	20.8 ± 5.3	22.1 ± 8.7	22.2 ± 7.8
ALT	19.0 ± 8.6	17.6 ± 6.7	19.0 ± 10.9	18.2 ± 9.4
ALP	55.0 ± 17.2	54.3 ± 16.4	53.8 ± 16.2	49.7 ± 12.1*^,†^
ɣ-GT	24.5 ± 23.6	24.3 ± 23.8	18.8 ± 18.7	18.5 ± 17.2
Total bilirubin	0.7 ± 0.3	0.7 ± 0.3	0.6 ± 0.2	0.6 ± 0.3
Fibrinogen	282.9 ± 68.6	286.2 ± 55.8	278.0 ± 68.6	288.0 ± 51.6*
Total cholesterol	194.0 ± 31.6	192.3 ± 32.0	202.3 ± 45.7	195.4 ± 31.7*
Triglycerides	107.4 ± 62.1	104.6 ± 51.1	104.4 ± 59.2	118.4 ± 98.1*
HDL-C	57.9 ± 10.0	65.4 ± 16.6	59.9 ± 9.8	70.5 ± 16.1*^,†^
LDL-C	116.0 ± 29.3	116.0 ± 32.0	119.7 ± 33.3	114.6 ± 28.8*
FBS	91.3 ± 13.5	90.8 ± 15.1	89.2 ± 10.3	88.7 ± 9.1
Insulin	6.1 ± 2.3	5.5 ± 3.7	5.5 ± 3.1	5.3 ± 3.4
HbA1c	5.6 ± 0.5	5.6 ± 0.6	5.4 ± 0.5	5.5 ± 0.5
HOMA-IR	1.4 ± 0.6	1.3 ± 1.0	1.3 ± 0.8	1.2 ± 0.8
eGFR	89.8 ± 16.1	85.2 ± 13.9	87.2 ± 13.4	86.6 ± 14.0
Uric acid	4.3 ± 0.8	4.2 ± 0.9	4.3 ± 0.9	4.3 ± 0.8
CRP	0.08 ± 0.09	0.11 ± 0.24	0.10 ± 0.14	0.12 ± 0.01

Data are presented as mean ± standard deviation.

*P < 0.05 based on paired *t* test, within each treatment; ^†^*P* < 0.05 based on *t* test between the two treatments.

*MHT* menopausal hormone therapy, *AST* aspartate aminotransferase, *ALT* alanine aminotransferase, *ALP* alkaline phosphatase, *ɣ-GT* gamma-glutamyltransferase, *HDL-C* high-density lipoprotein cholesterol, *LDL-C* low-density lipoprotein cholesterol, *FBS* fasting blood sugar, *HbA1c* hemoglobin A1c, *HOMA-IR* homeostatic model assessment for insulin resistance, *eGFR* estimated glomerular filtration rate, *CRP*,C-reactive protein.

**Figure 3:**
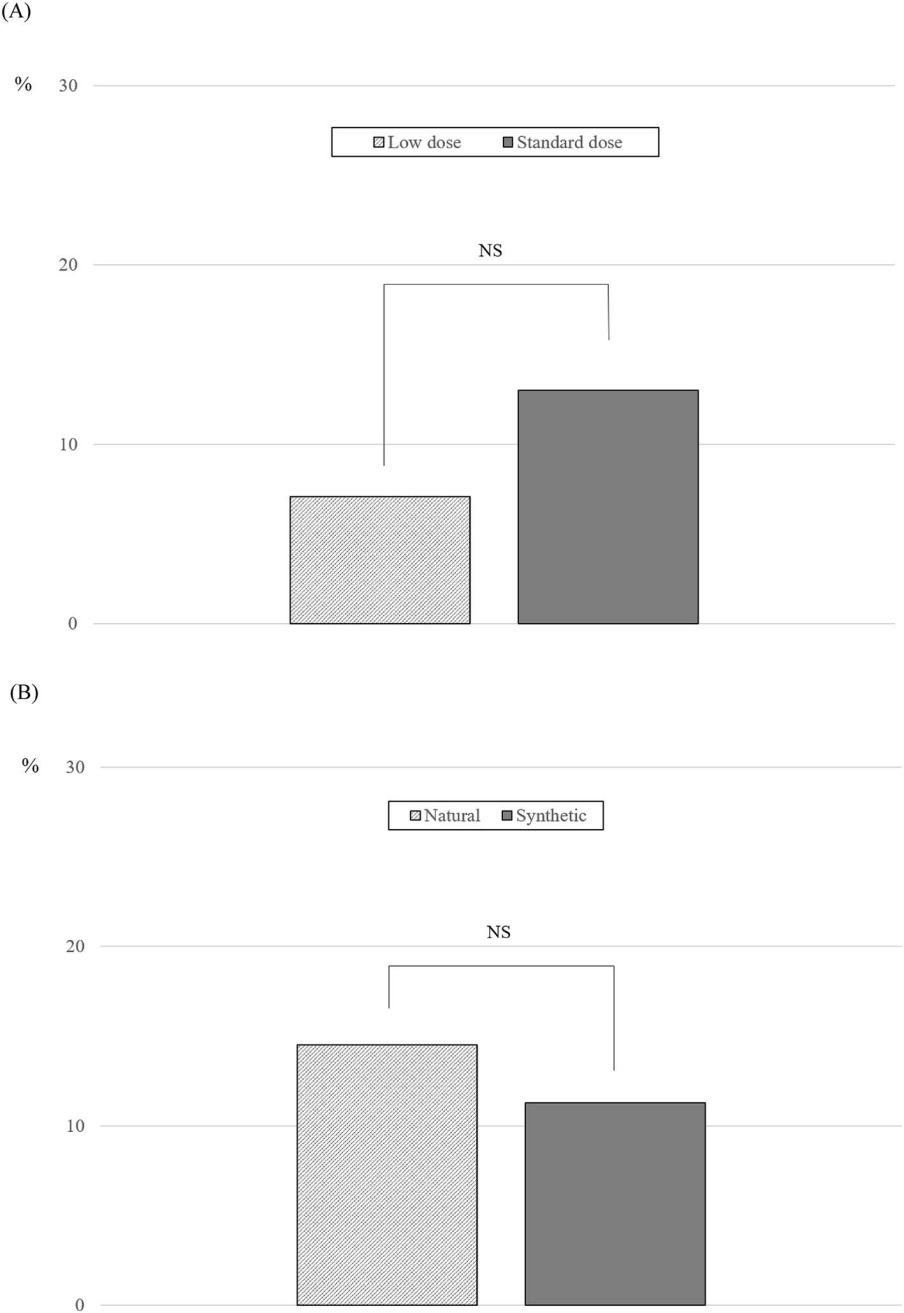
The proportion of progression (newly diagnosed or worsened severity) in non-alcoholic fatty liver disease (NAFLD) after menopausal hormone therapy (MHT) according to estrogen dose (**A**) and type of progestogen (**B**). *P* value based on chi-square test.

In addition, when the oral MHT group was divided into subgroups based on the dose of estrogen and type of progestogen, difference was not found in the progression of NAFLD between low-dose and standard-dose MHT (7.1% [6/85] vs. 13.0% [27/208], *P* = 0.146; Fig. 3A) or between natural progesterone and synthetic progestogen (14.5% [11/76] vs. 9.9% [18/181], *P* = 0.295; Fig. 3B).

## Discussion

In this retrospective cohort study, the effects of 12 months of MHT on the prevalence of NAFLD were compared based on the route of estrogen administration. Results showed transdermal estrogen can be more beneficial than oral estrogen for preventing the development or progression of NAFLD in postmenopausal women.

Estrogen affects the development or progression of NAFLD in various ways. Estrogen exerts anti-steatotic effect in hepatocytes and an anti-inflammatory effect in Kupffer cells. Estrogen also shows anti-fibrotic effects in hepatic stellate cells^22,23^. Overall, experimental data regarding the effects of estrogen on the liver are favorable toward inhibiting the development or progression of NAFLD. In addition to the direct effects of estrogen on the cells in the liver, estrogen has beneficial effects on lipid metabolism^17,24^. By improving dyslipidemia, which plays an important role in the pathogenesis of NAFLD, estrogen can indirectly contribute to preventing the development or progression of NAFLD. However, the significance of MHT on NAFLD has not been confirmed in human studies. Clinical studies for evaluating the effects of MHT on NAFLD diagnosed using sonography or liver biopsy remain limited and controversial, and most were cross-sectional studies. In a cross-sectional study including postmenopausal women in Brazil, the prevalence of NAFLD diagnosed with ultrasound was lower in women who received MHT than in women who did not (26.4% vs. 39.9%)^25^. However, in a 1-year study in Japanese women without pre-existing NAFLD, difference was not found in the development of NAFLD between women who received MHT (5.3%) and women who did not receive MHT (6.1%)^26^.

To the best of our knowledge, this is the first clinical study in which the different effects of MHT on NAFLD were assessed based on the route of estrogen administration. Although transdermal estrogen was predicted to show different effects compared with oral estrogen due to the first-pass effects with oral MHT^27^, the effects of MHT on NAFLD based on the route of estrogen administration have not been compared to date. In the present study, the prevalence of NALFD was significantly lower in the transdermal MHT group than in the oral MHT group after 12 months of MHT, and the change in the prevalence of NAFLD was opposite between the treatment groups (from 24.0% to 17.3% in the transdermal MHT group and from 25.3 to 29.4% in the oral MHT group). Because little or no changes were observed in the clinical characteristics, including weight and waist circumference, as well as in laboratory results, including insulin resistance, in the transdermal MHT group, the difference in the prevalence of NAFLD based on the route of estrogen administration may be explained by the change in lipid profile after oral MHT. Consistent with previous studies^17,24^, triglycerides significantly increased after oral MHT, which was different from other favorable changes observed in total, high- and low-density lipoprotein cholesterol. Because high triglyceride level is strongly associated with NAFLD^16,28,29^, transdermal MHT, which has a neutral effect on triglycerides, resulted in the favorable effects on NAFLD compared with oral MHT in the present study. Because moderate-to-severe vasomotor symptoms are associated with higher prevalence of NAFLD after adjustments^30^, transdermal MHT should be considered for postmenopausal women with pre-existing NAFLD or with higher risk of developing NAFLD.

In addition, the effects of oral MHT on the progression of NAFLD were evaluated based on the dose of estrogen and type of progestogen for the first time. Although the effects of estrogen on the liver and lipid profile are dose-dependent^17,24^ and progestogens can affect lipids, these issues have rarely been investigated. In the present study, the dose of oral estrogen did not affect the progression of NAFLD, although progression of NAFLD was apparently more prevalent with a standard dose than a low dose. The lowest effective dose of estrogen is recommended for oral MHT, especially in women with pre-existing liver disease such as NAFLD and other chronic liver diseases, or those with risk factors associated with liver disease. However, in healthy postmenopausal women, who are the most likely to receive MHT, a standard dose of oral estrogen does not appear to negatively affect the liver in terms of NAFLD and can be used to relieve severe symptoms based on the results of this study. However, progesterone has been suggested to influence carbohydrate, lipid, and protein metabolism and cause fat^31^. In previous studies, co-administration of progestogen during MHT was shown to reduce the favorable effects of oral estrogen on lipids, although the net effect remained beneficial^17,24^. However, clinical data to prove the effect of progestogens on NAFLD remain limited^32^. In the present study, progression of NAFLD did not differ based on the addition of progestogen or type of progestogen. However, despite the absence of a difference, due to the different clinical profiles between natural and synthetic progestogens on other organs (e.g., risk of breast cancer), natural progesterone is preferred, especially when a higher dose is needed. Micronized progesterone and dydrogesterone, which have little or no effect on the lipid profile, may be preferred for women with dyslipidemia in need of a progestogen^33^. More clinical studies in which the focus is on NAFLD are warranted in the future to determine a conclusion regarding the associations between dose of estrogen or use of progestogen and NAFLD.

The present study had several limitations. First, due to the retrospective study design, bias may have existed. Randomized controlled studies are warranted to confirm the superiority of transdermal estrogen to oral estrogen in terms of NAFLD progression. Second, the number of women receiving transdermal estrogen was smaller than women receiving oral estrogen. However, all consecutive postmenopausal women who met the criteria during the study period were included for analyses, and a lower prevalence of women receiving transdermal estrogen may reflect the real word in which transdermal MHT is less common^34^. In addition, diet and exercise, which are considered preventive and therapeutic measures for NAFLD, were not evaluated, although lifestyle modification was routinely recommended for all women. However, younger postmenopausal women (mean age 54 years) who received MHT and routine annual checkups in the health promotion center likely maintained a similar lifestyle during the study period as evidenced by unchanged body mass index and waist circumference after 12 months of MHT in both groups.

In conclusion, the results showed that transdermal MHT is more beneficial than oral MHT in terms of preventing the development and progression of NAFLD. The findings can help healthcare providers and patients in decision-making regarding choosing the best MHT option, especially in postmenopausal women who already have pre-existing NAFLD or who are at high risk of developing NAFLD.

## Data Availability

The datasets generated during and/or analysed during the current study are available from the corresponding author on reasonable request.

## Abbreviations

MHT: Menopausal hormone therapy
NAFLD: Non-alcoholic fatty liver disease
